# Deciphering the Structural, Textural, and Electrochemical Properties of Activated BN-Doped Spherical Carbons

**DOI:** 10.3390/nano9030446

**Published:** 2019-03-16

**Authors:** Bridget K. Mutuma, Boitumelo J. Matsoso, Damilola Momodu, Kabir O. Oyedotun, Neil J. Coville, Ncholu Manyala

**Affiliations:** 1 Department of Physics, Institute of Applied Materials, SARChI Chair in Carbon Technology and Materials, University of Pretoria, 0028 Pretoria, South Africa; bridgetmutuma@gmail.com (B.K.M.); dymomodu@yahoo.com (D.M.); kabir.oyedotun@gmail.com (K.O.O.); 2 Molecular Sciences Institute, School of Chemistry, University of the Witwatersrand, 2050 Johannesburg, South Africa; boijo.matsoso@gmail.com (B.J.M.); neil.coville@wits.ac.za (N.J.C.)

**Keywords:** carbon spheres, boron and nitrogen co-doped carbons, activation, supercapacitor

## Abstract

In this study, the effect of K_2_CO_3_ activation on the structural, textural, and electrochemical properties of carbon spheres (CSs) and boron and nitrogen co-doped carbon spheres (BN-CSs) was evaluated. Activation of the CSs and BN-CSs by K_2_CO_3_ resulted in increased specific surface areas and I_D_/I_G_ ratios. From the X-ray photoelectron spectroscopy (XPS) results, the BN-CSs comprised of 64% pyridinic-N, 24% pyrrolic-N and 7% graphitic-N whereas the activated BN-CSs had 19% pyridinic-N, 40% pyrrolic-N and 22% graphitic-N displaying the effect of activation on the type of N configurations in BN-CSs. A possible BN-co-doping and activation mechanism for the BN-CSs is proposed. Electrochemical analysis of the electrode materials revealed that BN doping, carbon morphology, structure, and porosity played a crucial role in enhancing the capacitive behavior of the CSs. As a proof of concept, a symmetric device comprising the activated BN-CSs displayed a specific power of 800 W kg^−1^ at a specific current of 1 A g^−1^ within an operating cell potential of 1.6 V in a 3 M KNO_3_ electrolyte. The study illustrated for the first time the role of K_2_CO_3_ activation in influencing the physical and surface properties of template-free activated BN-CSs as potential electrode materials for energy storage systems.

## 1. Introduction

Recent advances in nanotechnology research have generated tremendous scientific interest in the application of nanomaterials in energy storage devices, especially supercapacitors. Supercapacitors (SCs) are high-power electrochemical devices with fast charge-discharge dynamics and long cycling stability [1]. They have been applied as backup units for uninterrupted power storage devices in aerospace, power grids and in electric vehicles [1]. The unique material properties of carbon nanostructures such as chemical inertness, good electrical conductivity, high surface area and tunable surface chemistry have made them the most common supercapacitor electrode materials [2,3]. As such, carbon-based nanomaterials such as graphene, carbon nanotubes, mesoporous carbons and activated carbons have been widely explored [4,5,6]. The energy storage process in carbon materials is solely based on the electrochemical double-layer capacitance (EDLC), which is linked to the electrode material surface area, morphology, electrical conductivity as well as the surface chemistry of the carbons [7]. The ability to modulate the surface area, structural, and morphological properties of carbons allow for the generation of EDLC materials with unique properties.

The morphological framework of spherical carbons such as carbon onions and carbon spheres (CSs) provides an accessible surface for charge storage; thus, the spherical carbons are seen to be promising supercapacitor electrode materials. These spherical carbons can be synthesized using hydrothermal carbonization [8], direct pyrolysis [9] and chemical vapor deposition (CVD) methods [10]. Additionally, a vertically aligned CVD reactor allows for the synthesis of smaller sized carbon nanospheres and the ability to vary the surface functionality of carbons by controlling the carbon source, gas pressures, flow rates, and reaction times. To improve the textural and structural properties of the carbon structure, approaches such as the introduction of micropores and mesopores as well as the modification of the surface functionality of carbons have been reported [11,12,13,14]. These approaches allow for more electrolyte ion penetration into the pores and facilitate the improvement of the surface wettability of these carbons to enhance their electrochemical performance in supercapacitors [11,12,15,16]. 

One of the ways to facilitate surface reactivity and wettability of carbons is by substitutional heteroatom doping [17,18,19,20]. The heteroatom doping can also tailor the electron-donor properties of carbons and subsequently enhance their electrochemical activity [21]. Common dopants used for substitutional doping include boron [17], nitrogen [18], sulfur [22] and phosphorus [23]. Boron and nitrogen are more suitable dopants due to their atomic sizes and masses being closest to that of carbon [24]. Boron has three valence electrons and acts as an electron acceptor while nitrogen has five valence electrons and acts as an electron donor; thus, boron and nitrogen can alter the electronic structure of carbon and influence its electrochemical properties [25]. Recently BN co-doping of zero- and two-dimensional carbons has yielded higher electrochemical performance in supercapacitors than either B or N single doping [19,20,26,27]. However, for non-porous spherical carbons, a BN doping strategy alone cannot effectively influence the textural and electrochemical properties of carbon. Thus, in addition to heteroatom doping, the creation of a porous carbon structure is needed to further improve the electrochemical capacitance of CSs.

Chemical activation is a commonly used approach to modify the surface area and activity of carbon-based electrodes [28]. The chemical activation of already synthesized carbon nanomaterials has been reported for biomass-derived carbons, carbon nanotubes, reduced graphene oxide and graphene [29,30,31]. More recently, the chemical activation of spherical carbons has been carried out using sodium hydroxide, zinc chloride, phosphoric acid and potassium hydroxide [32,33,34]. For example, Yang et al. [35] reported on the KOH activation of CSs obtained by a hydrothermal reaction of a triblock copolymer Pluronic F108 (PEO_132_-PPO_50_-PEO_132_) template and phenolic resin as the carbon source. A specific capacitance of 182 F g^−1^ was recorded for the aggregated CSs at a specific current of 0.2 A g^−1^ with a capacitance retention of 70.5%. The high capacitance was ascribed to the hierarchical porous structure of the CSs (BET surface area: 1008 m^2^·g^−1^). However, the main drawback in the use of KOH was its complete destruction of the initial spherical carbon morphology [9,32]. While many researchers focus on the use of activated carbons as electrode materials for supercapacitors, the use of potassium carbonate (K_2_CO_3_) activated spherical carbons has been rarely explored. A recent approach of using K_2_CO_3_ as an activating agent has been reported by Sevilla et al. [36] and Moyo et al. [37]. Unlike other activating agents, the use of K_2_CO_3_ maintains the spherical morphology of carbon-based nanomaterials. The spherical carbons are attractive materials due to their consistent spheroidal geometry, chemical purity, and good chemical stability. Moreover, the presence of sp^2^ and sp^3^ carbon domains, dangling bonds, and the incomplete graphitic shells in CSs allow for high chemical reactivity and ease of surface modification. Thus, it is easy to modify the inner graphitic structure and the surface properties of CSs by the use of activating agents. However, reports on the use of CSs in electrochemical capacitors have focused on the activation of pristine carbon materials. One of the issues then is whether doped carbon materials will also maintain their morphology after activation.

While numerous reports have shown the importance of BN-doped carbon nanostructures in an electrochemical device using a template-assisted technique [20,38,39,40], the use of a template-free approach is requisite. Furthermore, most of the activated spherical carbons that are reported for application in supercapacitors, are largely generated from polymers and sucrose by hydrothermal reaction or pyrolysis methods [11,12,33,34]. These methods gave carbons with high surface areas. In contrast, CSs (doped and un-doped) synthesized by CVD technique have very low surface areas. Notwithstanding, the CVD method is still used commercially on an industrial scale to make carbon black for use in the tire industry [41]. Variations of this method to make CSs with different sizes/shapes [9] and with potential applications have been developed and attempts to enhance the surface areas of CSs have been explored [10,20]. 

Thus, a simple and template-free approach for the generation of chemically activated BN-doped carbons via a vertically oriented CVD reactor would be a useful approach for improving the electrochemical performance of doped carbons. The current work reports on the synthesis of BN-doped CSs by a simple post-doping process of CVD grown solid CSs in the presence of boric acid and urea. To achieve optimum electrochemical performance without compromising on the unique morphology of the carbon spheres, K_2_CO_3_ activation of the CSs and BN-CSs was explored. To our knowledge, this is the first time acetylene-derived BN-doped CSs have been activated with K_2_CO_3_ and applied in supercapacitors. This approach provided a synergistic effect of the activation as well as the influence of the heteroatom dopants that impacted on the carbon morphology, structure, and surface chemistry. A mechanism to elucidate the effect of activation on the surface chemical properties of CSs and BN-CSs is proposed. The K_2_CO_3_ activation of both CSs and BN-CSs resulted in the retention of their spherical shape, an excellent increase in the specific surface area (˃78 times) and consequently, a better electrode–electrolyte interaction. The activated BN-CSs symmetric cell in a 3 M KNO_3_ electrolyte solution exhibited a specific power of 2.4 kW kg^−1^ at a specific current value of 3 A g^−1^.

## 2. Experimental 

### 2.1. Starting Materials

Acetylene, C_2_H_2_ (99%, Afrox), argon, Ar (99%, Afrox), hydrogen, H_2_ (99.98%, Afrox) urea, CH_4_N_2_O (99%, Promark chemicals) and boric acid, H_3_BO_3_ (99.5%, Merck) were used for the synthesis of the CSs and BN-CSs. Potassium carbonate, K_2_CO_3_ (99.99%), potassium nitrate, KNO_3_ (99.99%), hydrochloric acid, HCl (37%), carbon acetylene black (99.95%), polyvinylidene difluoride, PVDF (99%) and N-methyl-2-pyrrolidone, NMP (99%), were purchased from Merck chemicals. Polycrystalline nickel foam (surface area of 420 m^2^·g^−1^ and 1.6 mm thickness, Alantum (Munich, Germany)) and microfiber filter paper (0.18 mm thickness, ACE chemicals) were used for the electrode preparation.

### 2.2. Experimental Procedure

The CSs were synthesized in a vertically oriented CVD reactor as reported elsewhere [42]. Boron and nitrogen co-doping of the pristine CSs was performed in a horizontal CVD reactor for 1 h at 900 °C. Briefly, a mixture of 50 mg of pristine CSs, 900 mg of urea, and 75 mg of boric acid was loaded onto a Cu-foil boat, which was placed at the center of a quartz tube and finally inserted in a horizontal CVD furnace. The furnace was heated to 900 °C at a heating rate of 10 °C min^−1^ under 3 sccm H_2_ and 300 sccm Ar flow for 1 h to obtain the BN-CSs product. A small amount of H_2_ was used during the thermal treatment to assist in the removal of excess C adatoms via hydrogenation thereby facilitating the completion of the aromatic graphitic domains as well as the formation of cleaner spheres’ surface. Similar results indicating the importance of small amounts H_2_ have been reported for graphene and other carbon nanomaterials [42,43]. Moreover, the hydrogenation process generates defects on the CS surface for easy attachment of heteroatoms during the doping process. The BN-CSs were mixed thoroughly with potassium carbonate as the activating agent in a mass ratio of 1:3 (BN-CSs: K_2_CO_3_) and mixed with a few drops of deionized water to make a sludge mixture. Subsequently, the drying of the sludge mixture was done at 80 °C for 24 h in an oven. The solid product was placed in an alumina boat and heated to 800 °C at a ramping rate of 5 °C min^−1^ in a horizontal furnace and held under Ar flow for 1 h. The obtained product was purified using a 1 M HCl solution and rinsed with deionized water until a neutral pH was obtained. The purified product was dried at 100 °C for 12 h and referred to as activated BN-CSs. A similar activation procedure was followed for the CSs to obtain the activated CSs. The morphological, structural and thermal properties as well as the elemental composition of the samples were evaluated using transmission electron microscopy (TEM), Raman spectroscopy, thermal gravimetric analysis (TGA), powder X-ray diffraction (XRD), N_2_ physisorption (Brunner, Emmet and Teller; BET) and X-ray Photoelectron Spectroscopy (XPS) (See details in the supplementary information).

### 2.3. Electrochemical Characterization

The electrode materials were prepared by mixing the active material (80 wt.%) with conductive carbon acetylene black (10 wt.%) and polyvinylidene fluoride (PVDF, 10 wt.%). A few drops of N-methyl-2-pyrrolidone (NMP) were added to the mixture to form a slurry which was then coated onto nickel foam (1 cm × 1 cm) and dried at 60 °C for 12 h. The electrochemical performance of the electrodes was explored using a Bio-Logic VMP300 potentiostat (Knoxville TN 37, 930, USA) controlled by the EC-lab V 11.40 software in a three-electrode configuration. A glassy carbon counter electrode, Ag/AgCl reference electrode and the CSs, BN-CSs, activated CSs, and activated BN-CSs as working electrodes were used to perform the electrochemical measurements in a 3 M KNO_3_ electrolyte solution. The mass loading of the active material was calculated to be approximately 3.6 mg for all the electrode materials.

A symmetric device was assembled in a coin-cell type configuration with a microfiber filter paper separator and 3 M KNO_3_ aqueous electrolyte. The cyclic voltammetry (CV) and galvanostatic charge-discharge (GCD) measurements were investigated at different scan rates and specific current values, respectively. The electrochemical impedance spectroscopy (EIS) measurements were carried out in a frequency range of 10 mHz to 100 kHz at an open circuit potential. The specific capacitance for a half-cell was calculated from the discharge curve of the GCD plot using Equation (1) while the specific capacitance of a single electrode in a symmetric device was calculated from the discharge slope of the GCD plot using Equation (2) [44];

(1) $$C_{S}=\frac{I\Delta t}{m\Delta V}\;F\;g{}^{-1}$$

(2) $$C_{el}=\frac{4I\Delta t}{m\Delta V}\;F\;g{}^{-1}$$

The specific energy and corresponding specific power of the device were calculated according to Equations (3) and (4) [37];

(3) $$E_{S}=\frac{C_{el}{\left(\Delta V\right)}^{2}}{28.8}\;\mathrm{Wh}\;\mathrm{kg}{}^{-1}$$

(4) $$P_{S}=\frac{3600E_{S}}{\Delta t}\;W\;{\mathrm{kg}}^{-1}$$

where *m* is the total mass of the electrode material, *C_S_* if the specific capacitance for a half-cell, *C_el_* is the specific capacitance of a single electrode in a full-cell, *I* is the current applied, Δ*t* is the discharge time and Δ*V* is the potential window.

## 3. Results and Discussion

### 3.1. Morphological, Structural, and Textural Properties

**Figure 1:**
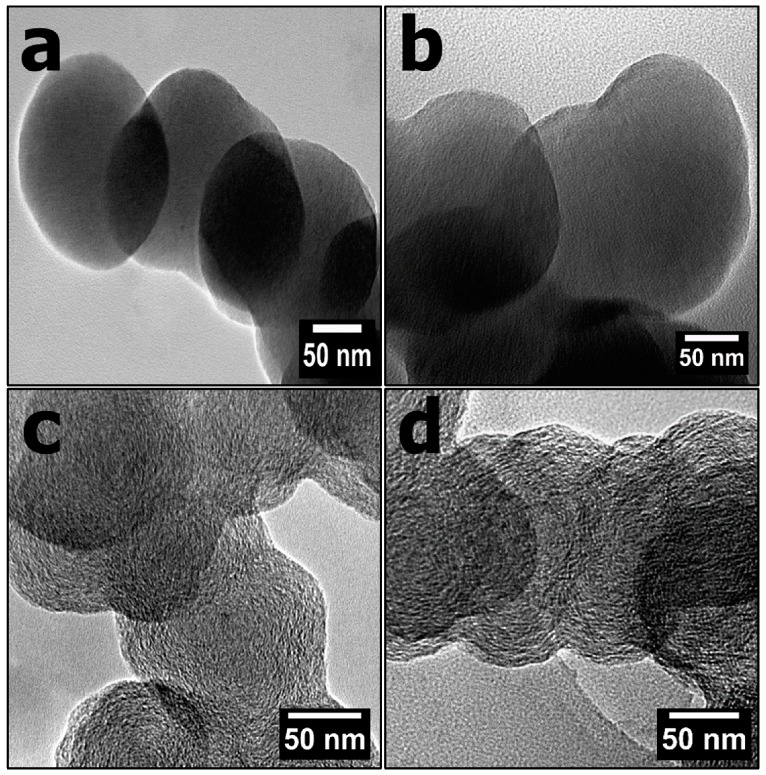
High magnification TEM images of (**a**) CSs, (**b**) BN-CSs, (**c**) activated CSs and (**d**) activated BN-CSs.

The TEM images revealed that the CSs had a spherical morphology (Figure 1a) and were accreted [45]. The BN-CSs, on the other hand, appears to have coalesced and had diameters (180 ± 12 nm) comparable to that of the pristine CSs (175 ± 11 nm) (Figure 1b). The small diameters for the CSs can be associated with the short dwell time used [42]. Typically, the growth of the spherical carbons is influenced by the type of precursor, reaction time, reaction temperature and the type of carrier gases [9,10,46]. Upon activation with K_2_CO_3_ at 800 °C, the particle size did not change (182 ± 16 nm) and the activated CSs and activated BN-CSs were accreted (Figure 1c,d). The accretion of the CSs can be explained using the diffusion-limited cluster aggregation model such as carbon black [47]. However, carbon black is synthesized by thermal decomposition of hydrocarbons at higher temperatures (>1300 °C) [41] or pyrolysis of used tires [48] and thus, is more electrically conductive and differ in nanostructure [46] from the CSs that are grown at lower temperatures (˂1100 °C). Moreso, the CSs mainly comprise of sp^2^ and sp^3^ domains and are classified as soot-like carbon particles. At higher temperatures, acetylene gas decomposes to give carbon and hydrogen radicals that nucleate to form pentagonal carbon rings [49]. The pentagonal carbon rings then undergo spiral shell growth followed by the nucleation of graphitic flakes due to the pairing of the heptagonal-pentagonal carbon rings to form the spherical carbon particles [50]. Finally, the van der Waals forces allow for random interconnection of the CS particles in different directions yielding an accreted network. During the activation process, the CSs surface reacted with K_2_CO_3_ to form CO resulting in the creation of edge defects on the surface and a distorted spheroidal geometry [36,51]. In contrast, the activated BN-CSs displayed a unique carbon morphology compared to both the pristine and activated CSs counterpart. This can be ascribed to the presence of the B and N atoms within the carbon matrix during activation leading to major differences in the CS surface. Further analysis of the activated BN-CSs surface showed a core and a disordered shell as well as the presence of a multicentered concentric onion-like structure (Figure S1). The interconnected carbon spheres suggest that the activated CSs and BN-CSs are suitable for electrochemical application as they could allow for better charge accumulation on the fused CS sphere surface and therefore, enhance the charge storage [13,52]. 

**Figure 2:**
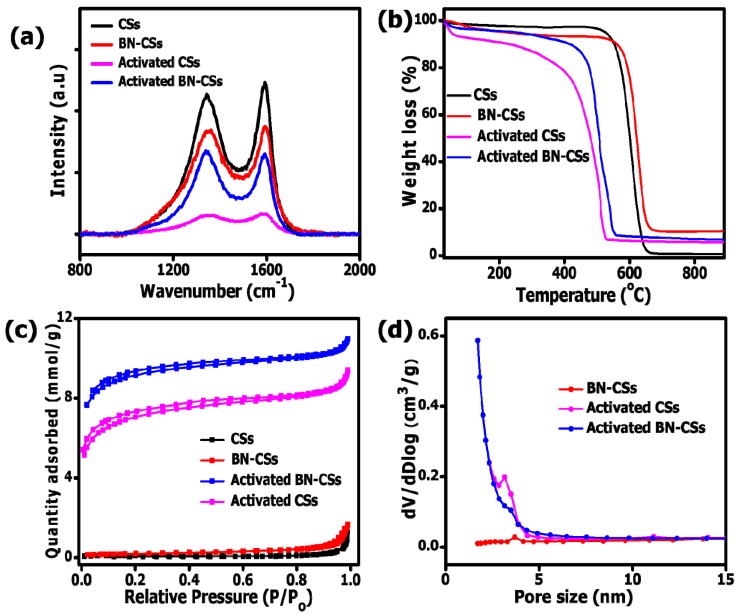
(**a**) Raman spectra, (**b**) thermogravimetric curves, (**c**) N_2_-adsorption-desorption isotherms and (**d**) pore-size distribution plots of the pristine and activated CSs and BN-CSs.

In Figure 2a, the Raman spectra of all as-synthesized materials exhibited spectral signals corresponding to the defect-induced D band (1344–1354 cm^−1^) and the graphitic-G band (1590–1598 cm^−1^) [53,54,55]. The lower D band position for the pristine CSs samples (~1344 cm^−1^), suggested a decreased relative concentration of the aromatic rings within the carbon lattice [56]. As expected, upon co-doping with boron and nitrogen atoms, an increase in the relative intensity of the D band was observed indicating distortion in the carbon lattice. More interestingly, a blue shift of the defect-induced D band (~1354 cm^−1^) was observed in the BN-CSs showing that heat treatment of the pristine CSs at 900 °C does not only incorporate B and N atoms into the carbon lattice but also affects the number of defects on the CSs [57,58]. Upon activation of the CSs and BN-CSs, a downshift in the D band was observed which is indicative of the decrease in the aromatic nature of the carbon lattice [56]. 

The defects in the pristine CSs, BN-CSs, activated CS and activated BN-CSs were deduced by calculating the ratio of the relative intensities of the D peak to that of the graphitic-G peak (I_D_/I_G_) [53,54]. From Table 1 it can be observed that doping with boron and nitrogen atoms increased the defect density ratio (I_D_/I_G_; 0.97) value in comparison with the moderate degree of graphitization of the CSs (i.e., I_D_/I_G_ ≈ 0.91). This can be attributed to the presence of more defects associated with the incorporation of B and N atoms into the carbon structure [58,59]. A shift in the G bands of activated CSs and BN-CSs with respect to the non-activated CSs can be ascribed to structural defects [60] incorporated in the carbon matrix resulting in an increase in the I_D_/I_G_ ratio values to 0.98 and 1.04, respectively (Table 1). 

Figure 2b shows the thermogravimetric analysis curves of the as-synthesized materials. The derivative profiles of the CSs are shown and discussed in the Supplementary Section (Figure S2a). The onset decomposition temperatures for the CSs and BN-CSs were at 504 °C and 534 °C with the complete decomposition of the carbon occurring at 664 °C and 680 °C, respectively. The slight change in the thermal stability of the CSs after BN co-doping can be associated with the incorporation of B and N heteroatom on the carbon nanostructure in agreement with the Raman data. For the activated CSs and BN-CSs, the onset decomposition temperature took place below 410 °C indicative of the loss of amorphous carbon domains emanating from the sp^3^ hybridized carbons and the disordered carbons created during the activation process [61]. The complete oxidation of the carbon in the activated CSs and activated BN-CSs occurred at 531 °C and 560 °C, respectively (Table S1). The lower onset decomposition peak for the activated CSs and activated BN-CSs indicates that the activated carbons are less thermally stable than the CSs further corroborating the results obtained from Raman analysis. This can be ascribed to the defects created on the carbon surface during activation as well as the presence of B and N atoms yielding more disorder on the carbon structure. The powder X-ray diffraction pattern of the synthesized CSs displayed two peaks at 2θ values of 24° and 44° characteristic of the (002) and (100) diffraction planes of graphite [9] (Figure S2b; detailed information given in the supplementary section).

Surface area and pore-size distribution of carbon-based electrode materials are essential properties due to the nature of energy storage which occurs via charge accumulation at the electrode–electrolyte interface. Figure 2c displays the N_2_ adsorption-desorption isotherms of the as-prepared carbon materials. The N_2_ isotherms of CSs and BN-CSs display type I typical isotherms which correspond to relatively non-porous materials (Figure 2c). The N_2_ sorption isotherm of activated CSs and BN-CSs illustrated narrow hysteresis loops at low relative pressures which is characteristic of materials containing micropores as well as capillary condensation observed in mesoporous materials [22,62,63,64]. The pore-size distribution plot (Figure 2d) confirmed the presence of micropores and mesopores in the activated samples with the activated BN-CSs having a high volume of pores. This accounts for the high specific surface area recorded. This indicated successful activation of the CSs and BN-CSs. Evidence of mesopores (2–5 nm) was also observed in the pore-size distribution plot and no macropores were detected. 

Table S2 gives a summary of the BET surface area and the pore-size distribution data of the CSs, BN-CSs, activated CSs and BN-CSs from the N_2_ adsorption-desorption measurements. The CSs displayed a surface area of 6.3 m^2^·g^−1^ implying a relatively non-porous material nature [42]. Upon co-doping with BN, the surface area increased slightly to 15.4 m^2^·g^−1^, due to a restructuring of the carbon matrix during the incorporation of the B and N atoms. Upon activation at elevated temperatures, the BET surface area of the activated CSs and BN-CSs drastically increased to 471 and 529 m^2^·g^−1^, respectively. Furthermore, the surface area of the activated BN-CSs was higher than that reported for polymer-derived BN-doped carbons (180 m^2^·g^−1^) [65], activated boron nitride (168 m^2^·g^−1^) [66] and BN-co-doped porous carbon nanotubes (79.8 m^2^·g^−1^) [67]. It is quite evident that activating the CSs and BN-CSs resulted in a tremendous increase in specific surface area (˃78 times) and increased porosity of the carbon structure. This can be ascribed to the emission of CO_2_ gas during the heat treatment; thus creating pores within the carbon framework and consequently leading to the formation of the porous carbon structure [36]. In addition, the total pore volume of the activated CSs and BN-CSs was observed to be much larger than that of their non-activated counterparts. 

### 3.2. Compositional Analysis Using XPS

**Figure 3:**
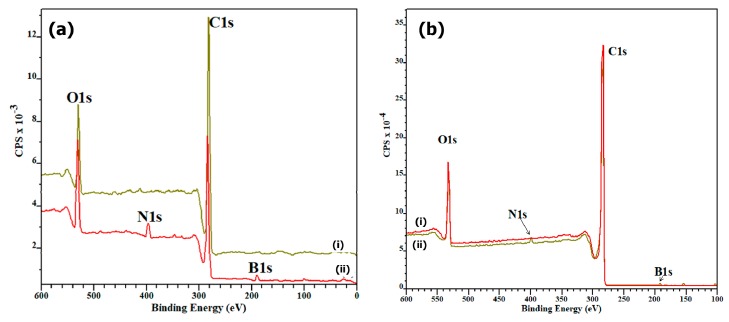
XPS survey spectra of (**a**) pristine CSs (**i**) and BN-CSs (**ii**); and (**b**) activated CSs (**i**) and activated BN-CSs (**ii**), respectively.

The elemental compositions of both pristine and activated CSs and BN-CSs were determined using XPS. The survey scans (Figure 3) showed two distinct peaks corresponding to C1s (283.9 eV) and O1s (531.1 eV) for all samples, as well as two small peaks corresponding to B1s (190.3 eV) and N1s (398.1 eV) for the survey spectra of the BN-CSs samples. The integral peak areas of the survey scans led to the calculation of the relative atomic percentages (at.%) of each component element within the samples, as illustrated in Table S3. The N and B content in the BN-CSs was 4.7 and 5.9 at.% while that in the activated BN-CSs was 1.0 and 1.5 at.%, respectively (Table S3). This drastic drop in the B and N content upon activation indicates removal of oxygenated nitrogen and boron atoms at the high temperatures [68]. The relative atomic percentage of B was found to be larger than that of the N content for the BN-CSs and activated BN-CSs which was in good agreement with the TGA data (increased thermal stability of the BN-CSs as compared to their un-doped counterparts). 

**Figure 4:**
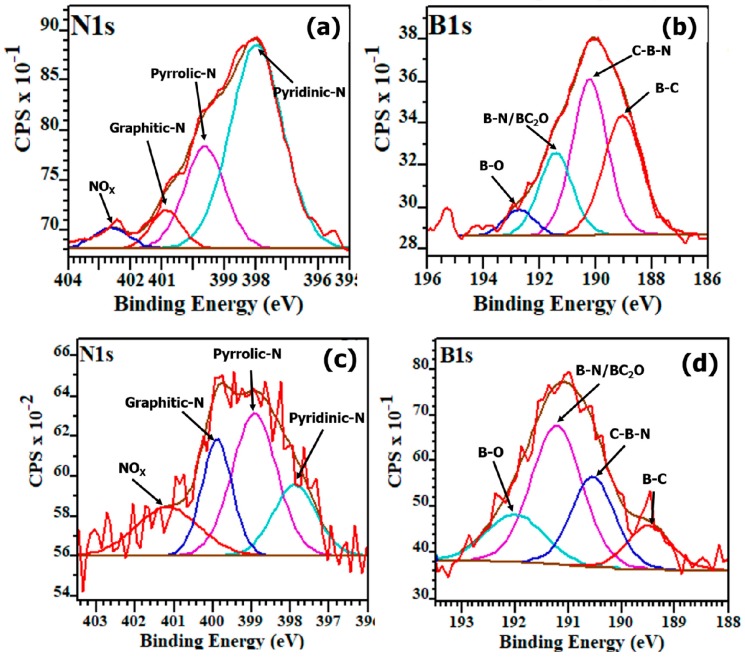
XPS spectra showing deconvoluted N1s and B1s peaks of the; (**a**,**b**) BN-CSs and (**c**,**d**) activated BN-CSs.

The deconvolution of the high-resolution XPS signals of C1s, O1s, N1s and B1s was used to determine the different chemical environments in the CSs and BN-CSs (Figures S3, S4, and Figure 4). From Figures S3 and S4, the XPS signal of the C1s was fitted to at least five components peaks. The main component peak (~284.0 eV) corresponds to the sp^2^ C=C bonds [69,70], whereas the peak located at ~284.8 eV can be ascribed to the presence of either sp^2^ C-N and/or C=N [71,72] bonds, as well as the presence of sp^3^ C bonds, such as sp^3^ C-C, sp^3^ C-H, and/or C-OH bonds [73]. Moreover, the lower binding energies at 282.7 eV, 282.9 eV 284.3 eV correspond to the formation of boron carbide bonds (B_4_C and/or BC_3_) [59,71]. Lastly, the peaks observed at 286.5 eV and 287.9 eV binding energies can be assigned to the oxygenated C atoms (C=O and N-C=O/O-C=O) in the disordered carbon structure [69,71,72].

The peak analysis of the N1s signal (Figure 4a,c) indicated the presence of four bonding states of nitrogen atoms, representing the sp^2^-N-B and/or pyridinic-N (398.0 eV), pyrrolic-N (399.6 eV), graphitic-N (400.9 eV), and oxygenated pyridinic nitrogen (NOx, 402.6 eV), respectively [59,72,74]. Due to the ambiguity associated with the analysis of the N1s XPS spectra for the chemical environments of both boron and nitrogen atoms, as a result of overlapping signals of h-BN and pyridinic domains, the B1s spectra were then used to establish the bonding states of B and N species. As a result, the B1s spectrum (Figure 4b,d) was deconvoluted into four component peaks centered at 189.0 eV, 190.2 eV, 191.4 eV, and 192.7 eV. The component peaks are ascribed to the B-C, BN/BC_2_O, sp^2^C-B-N and B-O bonds [59,75,76]. Finally, the O1s spectra (Figure S4b,d) was fitted to three peaks at 529.2 eV, 530.6 eV and 531.8 eV which were attributed to the O=C and/or O-N, O-C, and O-B bonds, respectively. Similarly, an overlap of component peaks was observed for the deconvolution of the O1s spectra; an overlap of the O=C-O with O-N (529.2 eV) as well as that of the O-B with O-C-O (531.8 eV) was observed.

**Scheme 1:**
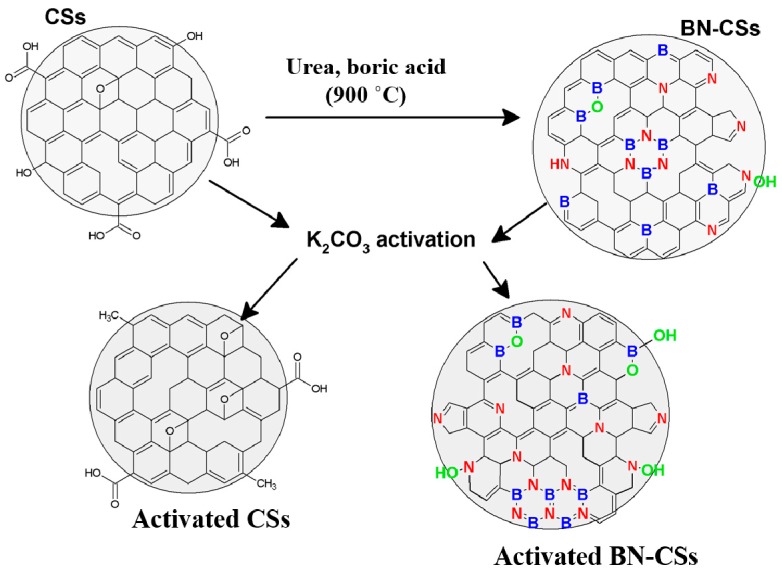
Proposed mechanism for the boron and nitrogen co-doping and activation of the CSs.

The average % concentrations for the various atoms and their relative bonding configurations were determined to give insight on the CSs doping and activation mechanism (Scheme 1). Boron and nitrogen co-doping of the CSs resulted in a decrease in the sp^2^C=C bonds and an increase in the sp^3^ C-C % concentrations (Table S4). This can be attributed to the incorporation of B and N inside the CSs matrix resulting in the formation of C-B, C-B-N, B-N, and B-O bonds. On the other hand, activation of the CSs yielded an increase in the sp^3^ C-C and C=O % concentrations. Typically, during activation of CSs, decomposition of K_2_CO_3_ and its reaction with carbon atoms results in the evolution of CO_2_ and CO gases, respectively [36,51]. This not only generates pores in the carbon matrix but also results in the creation of defect sites in the form of sp^3^ C-C and O-C-O bonds. Thus, the activated CSs possessed lower sp^2^C=C % concentrations as compared to their pristine counterparts. In contrast, the activation of BN-CSs impacts on their type of N and B configurations. In the activation process, some of the boron and nitrogen atoms attached to the carbon matrix leave the carbon surface as additional gaseous products. This results in fewer pyridinic-N and C-B % concentration creating more edge defects on the BN-CSs surface in the form of oxygenated NOx and B-O. Simultaneously, a rearrangement of the boron, carbon and nitrogen atoms takes place at high temperatures to give more pyrrolic-N, graphitic-N, and B-N configurations.

From the calculated average % concentrations for each N-configuration, the BN-CSs comprised of 64 ± 2% pyridinic-N, 24 ± 1% pyrrolic-N and 7 ± 1% graphitic-N whereas the activated BN-CSs had 19 ± 1% pyridinic-N, 40 ± 2% pyrrolic-N and 22 ± 1% graphitic-N, respectively (Table S4). The high percentage of graphitic-N and pyrrolic-N in the activated BN-CSs can be ascribed to the rearrangement of the carbon and nitrogen atoms within the carbon lattice during the activation process (Scheme 1). This further agrees with the increase in the average sp^2^ C=C % concentration observed for the activated BN-CSs showing that majority of the graphitic carbon present in the spherical carbon core was retained while the defect-induced carbon atoms attached to the pyridinic-N on the edges were removed during the activation process. Moreover, the NO_X_ concentration in the BN-CSs was lower (5 ± 1%) than that in the activated BN-CSs (19 ± 1%) indicating the presence of more defects on the edges of the activated BN-CSs. Furthermore, a reduction in the C-B % concentration was observed after activating the BN-CSs indicating the loss of boron-containing groups during the activation process. This resulted in the creation of structural defects while generating a porous carbon structure of the activated BN-CSs. Thus, corroborating the data from TEM, Raman, TGA, and BET measurements. In summary, XPS data analysis indicated the formation of different bonding configurations for carbon, boron, and nitrogen atoms (Tables S4 and S5). The negatively charged pyridinic-N, pyrrolic-N, B-C and O-C/B/N groups present on the CSs could readily influence their energy storage performance [75,77].

### 3.3. Electrochemical Analysis 

**Figure 5:**
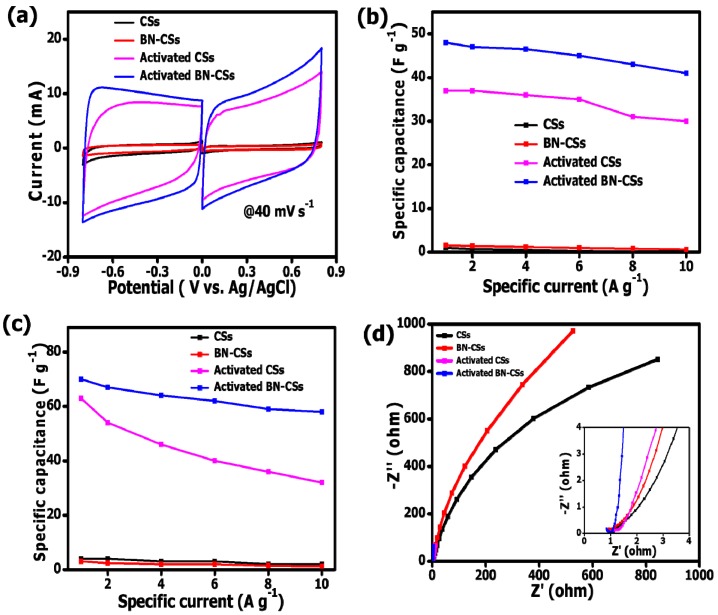
Pristine and activated CSs and BN-CSs: (**a**) CV curves at a scan rate of 40 mV s^−1^, and (**b**,**c**) specific capacitance calculated versus specific current for both positive and negative potential windows and (**d**) Nyquist plots in 3 M KNO_3_ electrolyte.

Figure 5 shows the results from the electrochemical analysis of the CSs electrodes in both positive and negative operating potential range in a neutral electrolyte solution, 3 M KNO_3_. The corresponding CV curves of the CSs, BN-CSs, activated CSs, and activated BN-CSs exhibited a quasi-rectangular shape signifying a characteristic electric double-layer capacitance behavior [1] (Figure 5a). As expected, the CV curve for the BN-CSs was more rectangular in shape than that of the CSs, thus displaying a less resistive carbon surface. The low current responses observed for the CSs and BN-CSs materials is ascribed to the lack of a porous structure (BET surface area ˂ 16 m^2^·g^−1^) that limited the ion diffusion and transportation between the electrode and electrolyte interface. On the contrary, the current response of the activated CSs and BN-CSs was higher due to the creation of pores on the carbon surface, which then facilitated a better electrolyte-electrode surface interaction and an enhanced ion diffusion into the pores [76,78]. Compared to the un-doped CSs, the activated BN-CSs gave the highest current response owing to their high surface area and the presence of B, N, and O heteroatoms. The activated CSs and BN-CSs electrodes were analyzed further by varying the scan rate and the specific current at a working potential of −0.8 to 0.0 V (Figure S5). 

The specific capacitance values for the CSs, BN-CSs, activated CSs, and activated BN-CSs, as calculated from the discharge slope of the GCD profiles, were 2, 2, 37 and 48 F g^−1^, respectively at a specific current of 1 A g^−1^ at a working potential of 0.0 to 0.8 V (Figure 5b). This remarkable increase in the specific capacitance can be attributed to the increase in porosity of the carbon surface and the BN co-doping. Similarly, a variation of the specific capacitance of all CSs was calculated from the discharge profile of the GCD plot in the operating potential range of −0.8 V to 0.0V and as observed, much higher capacitance values were obtained (Figure 5c). The specific capacitance values were calculated to be 5, 3, 62 and 70 F g^−1^ at 1 A g^−1^ for the CSs, BN-CSs, activated CSs, and activated BN-CSs, respectively, at a working potential of -0.8 to 0.0 V. Even though the specific capacitance value for the BN-CSs was slightly lower than that of the CSs, the CV curve of the former was more symmetric indicating an ideal EDLC behavior (Figure 5a). Besides, the interconnected carbon network in the activated BN-CSs could aid in ion diffusion. Indeed, the presence of non-spherical hollow cavities in carbon nanomaterials has been reported to reduce the charge transport pathway in electrochemical devices [79]. Moreover, the capacitance recorded for these activated BN-CSs was higher than that reported for CSs derived from polymeric precursors [80] and that of nanodiamond derived carbon onions [81] (Table S6).

The Nyquist plots show the impedance data of all fabricated sample electrodes in a 10 mHz to 100 kHz frequency range (Figure 5d). Basically, for an ideal capacitor, a vertical line which is parallel to the imaginary impedance axis should be observed at the low-frequency region. The real axis intercept constitutes the equivalent series resistance which is a sum of the resistance of the electrolyte, electrode and the contact resistance at the active material/current collector interface [82]. The CSs displayed a long diffusion length and a large deviation of the curve (black line) from the imaginary impedance axis at a low-frequency range corresponding to a non-ideal capacitance behavior (Figure 5d). After BN co-doping of the CSs, the steepness of the vertical line parallel to the imaginary Z-axis increased. This indicated a faster diffusion of ions between the electrode and electrolyte interface and less resistance between the electrode/electrolyte and the current collector. Upon activation of the CSs and BN-CSs, there was a significant decrease in the diffusion length with the activated BN-CSs portraying the shortest diffusion length. This indicated a better electrode–electrolyte interaction and a faster diffusion of the electrolyte ions in the activated CSs and BN-CSs. Additionally, this observation can indicate a higher hydrophilicity/surface wettability of the activated CSs and BN-CSs emanating from the presence of defect-induced carbon and the B and N heteroatoms after activation. Consequently, the equivalent series resistance values were 1.08, 0.96, 1.01 and 0.90 for the CSs, BN-CSs, activated CSs, and activated BN-CSs electrode materials, respectively. The lower equivalent series resistance values for the BN-CSs and the activated BN-CSs showed that indeed BN co-doping of the CSs increased the electronic conductivity and the charge transport between the electrode and the current collector [83]. 

The activated BN-CSs depicted a capacitive response closer to an ideal capacitance and a low resistance which is favorable for high power discharge applications. The improved capacitive properties of the activated BN-CSs electrodes can be ascribed to the interconnected carbon morphology that allowed for better charge accumulation on the carbon surface. This consequently improved the amount of charge stored. Moreover, the interconnected network could create a conductive network of the carbon matrix and consequently reduce the equivalent series resistance for the activated BN-CSs. Secondly, the disordered carbon structure of the activated BN-CSs characterized by the high I_D/_I_G_ ratio, and low thermal stability; caused high affinity for the aqueous KNO_3_ electrolyte ions creating better surface wettability as compared to the other carbons. Thirdly, the large pore volume and increased surface area allowed for easier accessibility of the electrolyte ions to the activated BN-CSs. Lastly, the presence of boron and nitrogen atoms, oxygenated functional groups, and the porous carbon surface had a synergistic effect on the carbon electrical conductivity, surface wettability, and, as a result, the overall amount of charge stored. 

**Figure 6:**
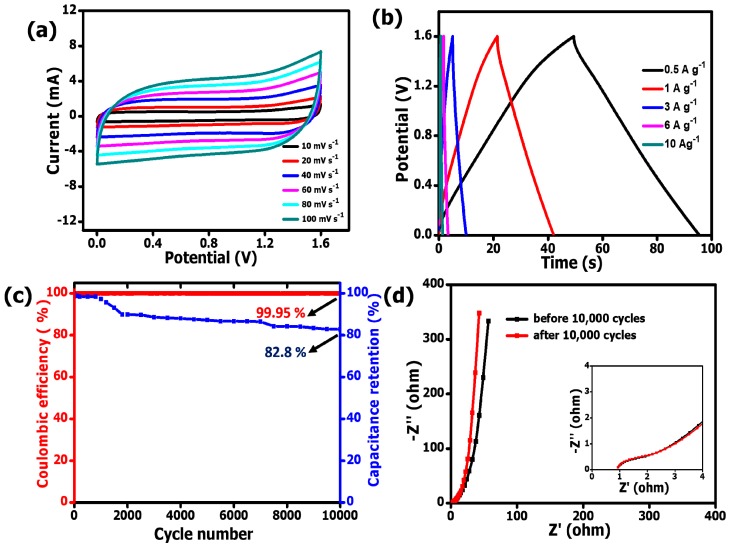
Electrochemical performance of the activated BN-CSs symmetric device showing (**a**) CV curves, (**b**) GCD plots, (**c**) Cycle stability and associated capacitance retention and (**d**) Nyquist plots before and after 10,000 cycles at 2 A g^−1^, respectively.

An operating cell potential of 1.6 V was chosen for the activated BN-CSs symmetric device and the CV profiles of the device at different specific currents are displayed in Figure 6a. Figure 6b shows the associated GCD profiles of the symmetric supercapacitor at increasing specific current values. A specific capacitance of 58 and 52 F g^−1^ was recorded for the device at 0.5 and 1 A g^−1^, respectively. This shows that over 80% of the initial capacitance was retained when the specific current was doubled. The capacitance values in this device are comparable to those reported in the literature for CSs. For instance, Kim et al. [52] reported a specific capacitance of 56 F g^−1^ at 0.58 A g^−1^ for monodispersed starburst CSs (1260 m^2^·g^−1^) in a two electrode system in a potential window of −0.5 V to 0.5 V in 1 M Na_2_SO_4_ electrolyte. They attributed the electrochemical performance of their device to the interconnected CSs morphology.

**Figure 7:**
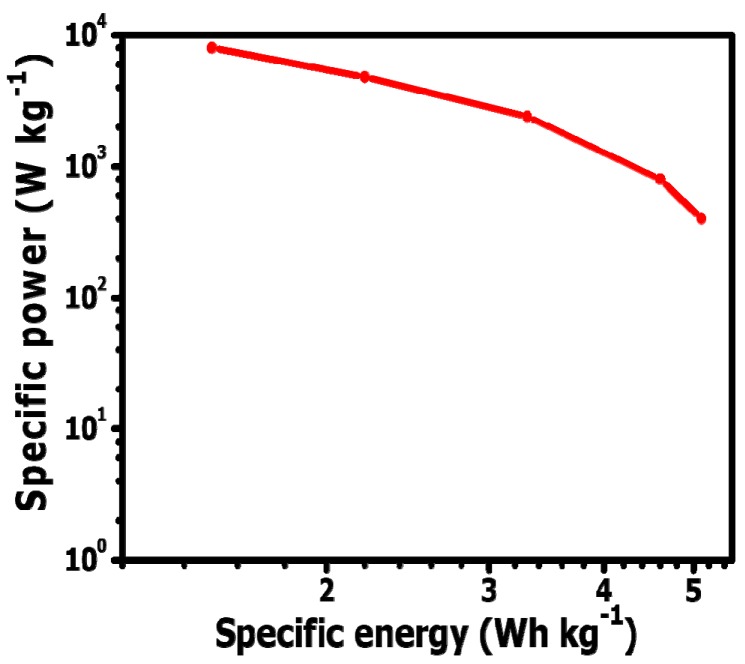
Ragone plot of the activated BN-CSs symmetric cell.

A cycling stability test was carried out on the symmetric device with continuous charging-discharging for up to 10,000 cycles at a specific current of 2 A g^−1^ (Figure 6c). A coulombic efficiency of 99.95% with a capacitance retention value of 83% was obtained for the device after 10,000 cycles. An EIS Nyquist plot before and after cycling showed a negligible change in the equivalent series resistance of the device (Figure 6d). This shows that the electrode–electrolyte interface and electrode resistance did not increase even after 10,000 cycles. Figure 7 shows the Ragone plot of the activated BN-CSs symmetric cell. The specific energy of the device was calculated as 4.6 Wh kg^−1^ with a corresponding specific power of 800 W kg^−1^ at a specific current of 1 A g^−1^. The specific energy at 3 A g^−1^ was 3.3 Wh kg^−1^ corresponding to a specific power of 2400 W kg^−1^. Despite the low specific energy, the relatively high specific power achieved was higher than values reported on other heteroatom doped spherical carbons and related materials (Table S6) [20,84,85,86]. For example, Zhang et al. [84] reported an energy density of 4 Wh kg^−1^ for solid CSs with a specific power of less than 200 W kg^−1^. The nitrogen-doped CSs with a surface area of 1229 m^2^·g^−1^ exhibited a specific energy of 7.67 Wh kg^−1^ with a corresponding specific power of 150.1 W kg^−1^. The authors attributed this observation to a combined effect of the high surface area as well as a high nitrogen content doping. The high specific power exhibited by the activated BN-CSs shows that the materials are applicable for use in high power demand systems. Moreover, the specific energy of the activated BN-CSs was higher than that reported for carbon nano-onions [86]. Nonetheless, it is to be noted that the structural and electrochemical properties of the activated BN-CSs (onion-like morphology) described in this study differ significantly with those of nanodiamond derived carbon onions. 

## 4. Conclusions

In this study, we reported on the preparation of BN-CSs using a simple post-doping step of CSs in the presence of urea and boric acid at 900 °C. The physicochemical properties of the carbons were modulated by activating the CSs and BN-CSs with an optimal ratio of K_2_CO_3_. Activation of the CSs resulted in an interconnected carbon morphology, a large increase in surface area and porosity besides the creation of disorder in the carbon. The BN-CSs were comprised of 5.9% B and 4.7% N whereas the activated BN-CSs had 1.5% B and 1.0% N indicating the effect of activation on the heteroatom composition within the CSs. The BN-CSs comprised of 64% pyridinic-N, 24% pyrrolic-N and 7% graphitic-N whereas the activated BN-CSs had 19% pyridinic-N, 40% pyrrolic-N and 22% graphitic-N, illustrating the effect of activation on the N-configurations in BN-CSs. The activated BN-CSs gave the highest surface area, longest discharge time, lowest equivalent series resistance and the highest specific capacitance, as compared to the other CSs electrode materials. Furthermore, from the EIS data, the activated BN-CSs exhibited the shortest diffusion path length indicative of a less resistive carbon surface and better ion diffusion compared to the activated CSs. A symmetric device fabricated from the activated BN-CSs material was functional within an operating cell potential of 1.6 V and exhibited a specific power of 800 and 2400 W kg^−1^ at specific current values of 1 and 3 A g^−1^, respectively. Moreover, the capacitance retention of the activated BN-CSs was 83% after 10,000 cycles at a specific current of 2 A g^−1^. In summary, we have demonstrated that K_2_CO_3_ activation of CSs and BN-CSs plays a significant role in modifying their morphological, structural, textural, and electrochemical properties. This study provides insight on the significant role of K_2_CO_3_ activation and boron and nitrogen post-synthesis doping on the surface chemistry and applicability of CSs in energy-related systems. 

## Figures and Tables

**Table 1:** The D and G band positions and I_D_/I_G_ ratio of the CSs and BN-CSs.

Material	D Band Position (cm^−1^)	G Band Position (cm^−1^)	I_D_/I_G_ Ratio
CSs	1344	1594	0.91
BN-CSs	1354	1590	0.97
Activated CSs	1343	1598	0.98
Activated BN-CSs	1340	1596	1.04

## Supplementary Materials

The Supplementary Materials are available online at https://www.mdpi.com/2079-4991/9/3/446/s1.
