# Supplementary material for: Deciphering the Structural, Textural, and Electrochemical Properties of Activated BN-Doped Spherical Carbons
Source: Nanomaterials (Basel). 2019 Mar 16;9(3):446. doi: 10.3390/nano9030446 (PMC6474088; doi:10.3390/nano9030446)
Supplement: Supplementary file 1 [file nanomaterials-09-00446-s001.pdf]

nanomaterials

MDPI

Article

Deciphering the Structural, Textural, and Electrochemical Properties of Activated BN-Doped Spherical Carbons

Bridget K. Mutuma [1,2], Boitumelo J. Matsoso [2], Damilola Momodu [1], Kabir O. Oyedotun [1], Neil J. Coville [2] and Ncholu Manyala [1,*]

[1] Department of Physics, Institute of Applied Materials, SARChI Chair in Carbon Technology and Materials, University of Pretoria, 0028 Pretoria, South Africa; bridgetmutuma@gmail.com (B.K.M.); dymomodu@yahoo.com (D.M.); kabir.oyedotun@gmail.com (K.O.O.)

[2] Molecular Sciences Institute, School of Chemistry, University of the Witwatersrand, 2050 Johannesburg, South Africa; boijo.matsoso@gmail.com (B.J.M.); neil.coville@wits.ac.za (N.J.C.)

* Correspondence: ncholu.manyala@up.ac.za; Tel.: +27-12-420-3549

Received: 1 February 2019; Accepted: 11 March 2019; Published: 16 March 2019

Supplementary Information

1. Characterization of the pristine and activated CSs and BN-CSs

The morphology of the CSs, BN-CSs, activated CSs and activated BN-CSs was determined by transmission electron microscopy (TEM) using a JEOL JEM-2100F electron microscope operating at 200 kV. The Raman analysis was carried out on a WITec Alpha 300R Plus confocal micro-Raman Spectrometer at room temperature with a laser excitation wavelength of 532 nm at 2.0 mW laser power. The thermal stability of all the CSs was investigated by thermal gravimetric analysis (TGA) on a SDT Q600 TGA instrument conjugated with a weight loss derivative curve. For each sample, about 5.0 mg was placed in a ceramic pan and loaded onto the instrument furnace. The sample was heated from 60 °C to 900 °C at a ramp rate of 10 °C min^{-1} under air flow. The percentage weight change over increasing temperature (TGA plot) provided information about the thermal stability of the samples. The powder X-ray diffraction (XRD) analysis was carried out on an XPERT-PRO diffractometer (PANalytical BV, Netherlands) with a radiation source (Co Kα, λ = 0.178901 nm). The details of the set-up parameters for testing the samples included a reflection geometry at 2θ values (18–90°) and at a step size of 0.01°. The N_2 adsorption and desorption isotherms of the CSs were measured at 77 K using a Micromeritics Tristar II 2020 pore analyzer. The samples were initially degassed at 180 °C for 12 h under vacuum condition. The specific surface area was calculated by the Brunner, Emmet and Teller (BET) method from the N_2-adsorption data. The Barett-Joyner-Halenda (BJH) method was used to determine the pore size distribution by analysing the adsorption-desorption branches of the N_2 isotherms. The chemical composition of the carbon nanomaterials was determined by X-ray Photoelectron Spectroscopy (XPS) using a Physical Electronics Quantum 2000. The monochromatised aluminium Kα radiation was used with a 200 µm spot size. An X-ray power of 50 W at a pressure of < 10^{-8} mbar was applied. The data analysis of the XPS spectra was conducted using the CasaXPS software and was fitted with Lorentzian-Gaussian (GL30) peaks.

2. Results

2.1. Morphological data

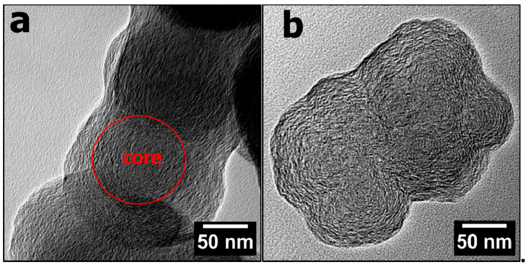

Figure S1. TEM image showing (a) core-shell morphology and (b) coalesced multi-centred onion-like structure of the activated BN-CSs.

2.2. TGA and XRD analysis

Table S1. TGA data for the CSs, BN-CSs, activated CSs and activated BN-CSs.

Material	Onset decomposition temperature (°C)	Decomposition peak centre (°C)	Residual weight (wt. %)
**CSs**	326, 504	602	< 0.4
**BN-CSs**	534	622	10.3
**Activated CSs**	268, 338, 370	509	5.5
**Activated BN-CSs**	290, 330, 402	473, 493, 530	7.0

The effect of the BN doping and activation on the thermal properties of the CSs was further studied in detail using TGA. The first order derivative plots showed that the pristine CSs had two decomposition temperatures (Figure S2a). The first weight loss peak is ascribed to the loss of amorphous carbon domains at ~ 326 °C while the second decomposition peak was due to the oxidation of carbons in air. For the BN-CSs, there is no weight loss peak observed at ~ 326 °C characteristic of a more graphitic carbon. The activated CSs and BN-CSs exhibited a weight loss peak at 268 and 290 °C; an indication of the possible presence of functional groups such as C=O and C-O-C on the surfaces of CSs. For the activated BN-CSs, multiple weight loss peaks were observed at 473, 493 and 530 °C as compared to their un-doped counterparts (Table S1). This is possibly due to the presence of various functional groups; B-C (448 kJ mol^{-1}), OB/B-O (536 kJ mol^{-1}), B-N (389 kJ mol^{-1}), and nitrogen bonded to sp^2 C and/or sp^3C (339 kJ mol^{-1} and 444 kJ mol^{-1}), that decompose at different temperatures. Furthermore, the core-shell morphology, as well as the coalesced multiple centers as observed in the TEM micrographs indicated that more structural deformation and/or disorder was induced by the activation of the BN-CSs. The residual weight (ash) after thermal gravimetric analysis in air was calculated to be < 0.4, 10.3, 5.5 and 7.0 wt.% for the CSs, BN-CSs, activated CSs and activated BN-CSs, respectively. The low residual weight for the pristine CSs confirmed the high level of carbon purity. After BN-doping more residue was left on the ceramic pan suggesting the formation of the thermally stable boric oxide [1]. An increase in the residue was recorded for the activated CSs suggesting the presence of potassium remnants. The residual weight of the activated BN-CSs was lower than that of the BN-CSs indicating the loss of oxygenated boron and nitrogen containing groups as was later confirmed from the XPS data.The powder X-ray diffraction pattern of the synthesized CSs displayed two peaks at 2θ values of 24° and 44° (Figure S2b). This was attributed to the (002) and (100) diffraction planes of graphite [2]. These peaks were broad in all of the samples indicating that the CSs

were amorphous in nature. However, an upshift of the (002) peak was observed after doping the BN-CSs indicating a change in the graphitic interlayer spacing due to B and N heteroatoms [3]. In the activated BN-CSs, additional peaks were observed at 19.5°, 31.5° and 38.5° indicating the presence of B_2O_3 and B_xC domains in the carbon matrix [4]. This showed that BN co-doping and activation affected the crystallinity of the CSs.

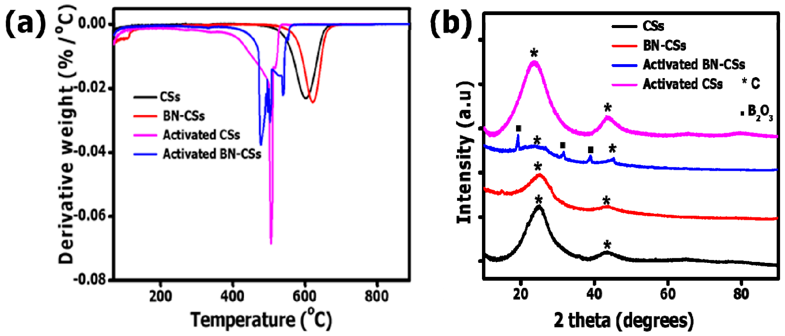

Figure S2. (a) Thermal gravimetric derivative profiles and (b) powder X-ray diffraction patterns of the samples.

2.3. Physisorption and XPS data

Table S2. Textural properties of carbon materials.

Sample	BET surface area (m^2·g^{-1})	V_{total} (cm^3·g^{-1})	V_{meso} (cm^3·g^{-1})
**CSs**	6	0.02	0.004
**BN-CSs**	15	0.02	0.017
**Activated CSs**	471	0.31	0.17
**Activated BN-CSs**	529	0.33	0.11

Table S3. Elemental composition of the carbon materials.

Material	Elemental composition (at %)			
	C	N	B	O
**CSs**	88.5	-	-	11.5
**BN-CSs**	73.7	4.7	5.9	15.7
**Activated CSs**	89.4	-	-	10.6
**Activated BN-CSs**	87.5	1.0	1.5	10.0

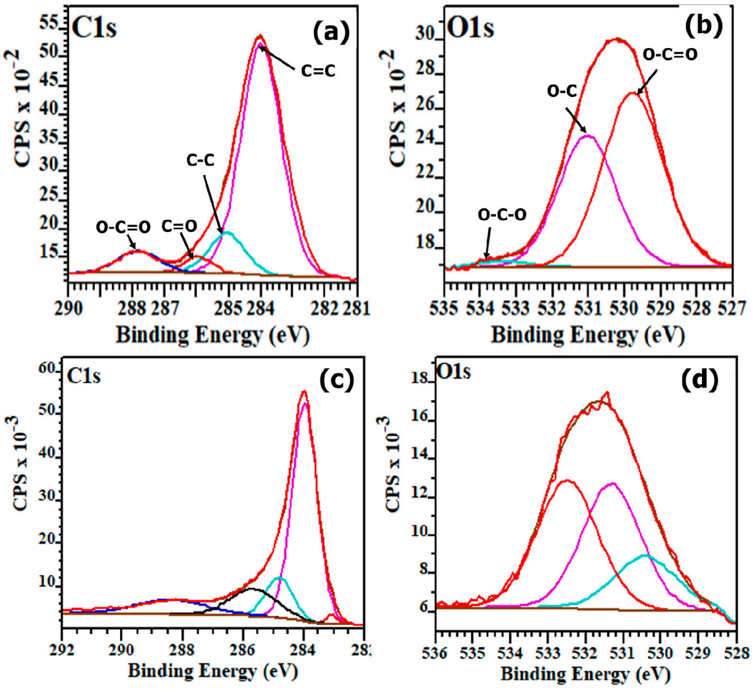

Figure S3. XPS spectra showing deconvoluted curves of the C1s and O1s in; (a and b) pristine CSs and (c and d) activated CSs.

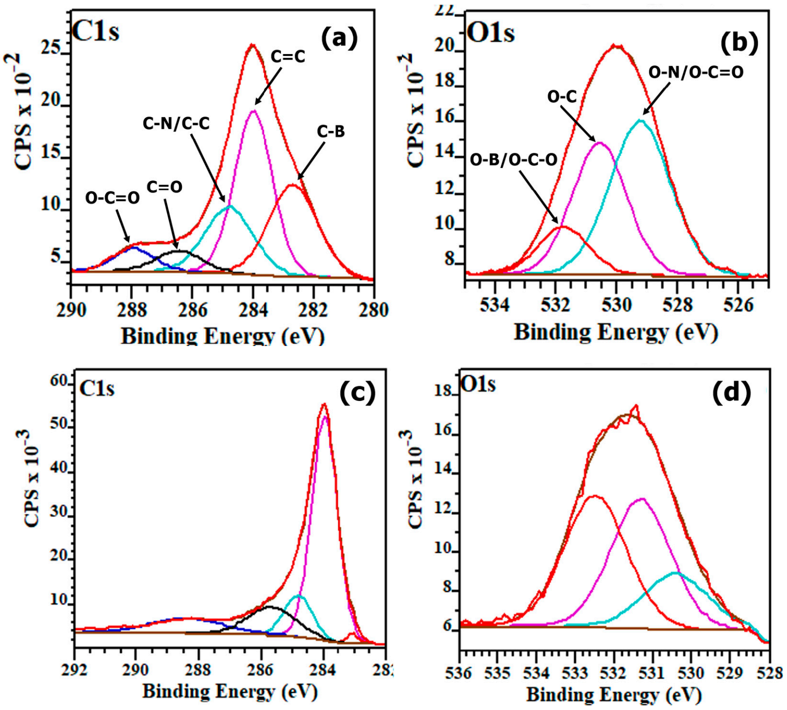

Figure S4. XPS spectra showing deconvoluted curves of the C1s and O1s in; (a and b) BN-CSs and (c and d) activated BN-CSs.

Table S4. Summary of % concentration from C 1s and N 1s spectra.

Material	Ave. % Concentration				
	C-B	sp^2 C=C	C-N/sp^3 C-C	C=O	O-C=O
CSs	-	77 ± 2	12 ± 2	4 ± 1	7 ± 1
BN- CSs	28 ± 2	39 ± 2	20 ± 1	7 ± 1	6 ± 1
Activated CSs	-	55 ± 2	24 ± 2	15 ± 1	6 ± 1
Activated BN- CSs	2 ± 1	60 ± 2	12 ± 1	14 ± 2	12 ± 2
	h-BN/ pyridinic-N		Pyrrolic-N	Graphitic-N	NOx
BN- CSs	64 ± 2		24 ± 1	7 ± 1	5 ± 1
Activated BN-CSs	19 ± 1		40 ± 2	22 ± 1	19 ± 1

Table S5. Summary of % concentration from B1s and O 1s spectra.

Material	Ave. % Concentration			
	B-C	C-B-N	B-N/ BC_2O	B-O
BN-CSs	34 ± 2	40 ± 2	20 ± 1	6 ± 1
Activated BN-CSs	12 ± 1	25 ± 1	45 ± 2	18 ± 2
	O-N/O=C-O	O-C	O-B/O-C-O	
CSs	62 ± 1	36 ± 1	2 ± 1	
BN-CSs	50 ± 2	38 ± 2	12 ± 1	
Activated CSs	23 ± 1	27 ± 1	50 ± 2	
Activated BN-CSs	18 ± 1	40 ± 2	42 ± 2	

2.4. Electrochemical data

The activated CSs and BN-CSs electrodes were analyzed further by varying the scan rate and the specific current values at a working potential of -0.8 to 0.0 V. The CV curve of the activated CSs was slightly distorted while that of the activated BN-CSs maintained a rectangular shape even at high scan rates of 100 $mV.s^{-1}$ (Figure S5a and S5c). This can be ascribed to a high surface area, reactivity and a less resistive behaviour of the activated BN-CSs electrode material. Figure S5b and S5d displays the GCD curves of the activated CSs and BN-CSs electrodes at different values of specific current. Both electrode materials displayed triangular curves at all specific currents without a significant IR drop. The discharge time for the activated BN-CSs was longer than that displayed by the activated CSs at all the specific currents indicative of a higher specific capacitance.

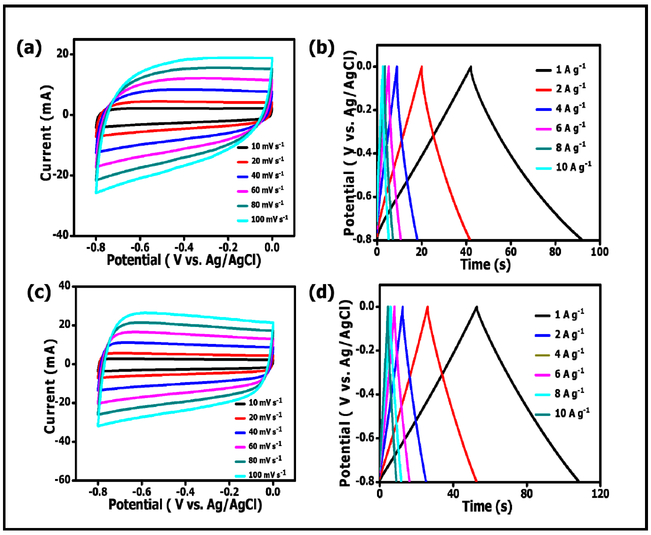

Figure S5. Cyclic voltammograms and GCD profiles of (a and b) activated CSs, and (c, d) activated BN-CSs in 3M KNO_3, respectively.

Table S6. Summary of specific capacitance, energy and power of CSs and related materials.

Material	Electrolyte	Specific capacitance (F g^{-1})	Specific energy (Wh kg^{-1})	Specific power (W kg^{-1})	Ref
**CSs**	**1 M H_2SO_4**	13.4	-	-	[5]
**N-CSs**		42.7			
**Solid carbon spheres**	**6 M KOH**	-	4	200	[6]
**BCN co-doped porous carbon**	**6 M KOH**	125	3	201	[7]
**MXenes**	**30 wt.% KOH**	51	3.4	700	[8]
**Pristine CNOs**	**2 M KNO_3**	25.8	1.5	123 000	[9]
**Annealed carbon onions**	**1.5 M NEt_4BF_4 in acetonitrile**	22.5	-	60 000	[10]
**Activated BN-CSs**	**3 M KNO_3**	52	4.6	800	**This work**

CSs; carbon spheres, N-CSs; nitrogen-doped carbon spheres, CNOs: carbon nano-onions; BCN: boron, carbon and nitrogen co-doped porous carbon, NEt_4BF_4: tetraethyl ammonium tetra-fluoroborate; BN-CSs; boron and nitrogen co-doped carbon spheres.

References

1. Z.N. Tetana, S.D. Mhlanga, N.J. Coville, Chemical vapour deposition syntheses and characterization of boron-doped hollow carbon spheres, Diam. Relat. Mater. 74 (2017) 70–80. doi:10.1016/J.DIAMOND.2017.02.005.
2. Y.Z. Jin, C. Gao, W.K. Hsu, Y. Zhu, A. Huczko, M. Bystrzejewski, et al., Large-scale synthesis and characterization of carbon spheres prepared by direct pyrolysis of hydrocarbons, Carbon N. 43 (2005) 1944–1953. doi:10.1016/J.CARBON.2005.03.002.
3. J. Pang, W. Zhang, H. Zhang, J. Zhang, H. Zhang, G. Cao, et al., Sustainable nitrogen-containing hierarchical porous carbon spheres derived from sodium lignosulfonate for high-performance supercapacitors, Carbon N. 132 (2018) 280–293. doi:10.1016/J.CARBON.2018.02.077.
4. A. Sinha, T. Mahata, B.. Sharma, Carbothermal route for preparation of boron carbide powder from boric acid–citric acid gel precursor, J. Nucl. Mater. 301 (2002) 165–169. doi:10.1016/S0022-3115(02)00704-3.
5. W. Lee, J.H. Moon, Monodispersed N-Doped Carbon Nanospheres for Supercapacitor Application, ACS Appl. Mater. Interfaces. 6 (2014) 13968–13976. doi:10.1021/am5033378.

6. A. Zhang, S. Cao, Y. Zhao, C. Zhang, A. Chen, Facile one-pot hydrothermal synthesis of particle-based nitrogen-doped carbon spheres and their supercapacitor performance, New J. Chem. 42 (2018) 6903. doi:10.1039/c8nj00576a.
7. H. Guo, Q. Gao, Boron and nitrogen co-doped porous carbon and its enhanced properties as supercapacitor, J. Power Sources. 186 (2009) 551–556. doi:10.1016/J.JPOWSOUR.2008.10.024.
8. R.B. Rakhi, B. Ahmed, M.N. Hedhili, D.H. Anjum, H.N. Alshareef, Effect of Postetch Annealing Gas Composition on the Structural and Electrochemical Properties of Ti_2CT_x MXene Electrodes for Supercapacitor Applications, Chem. Mater. 27 (2015) 5314–5323. doi:10.1021/acs.chemmater.5b01623.
9. Y. Gao, Y. Shen, M. Qian, X. Nan, J. Redepenning, P. Goodman, et al., Chemical activation of carbon nano-onions for high-rate supercapacitor electrodes, Carbon N. 51 (2012) 52–58. doi:10.1016/j.carbon.2012.08.009.
10. C. Portet, G. Yushin, Y. Gogotsi, Electrochemical performance of carbon onions, nanodiamonds, carbon black and multiwalled nanotubes in electrical double layer capacitors, Carbon N. 45 (2007) 2511–2518. doi:10.1016/j.carbon.2007.08.024.
